# Microstructural white matter alterations in dementia with lewy bodies and Alzheimer’s disease: a diffusion tensor imaging and neurite orientation dispersion and density imaging study

**DOI:** 10.3389/fnagi.2026.1830477

**Published:** 2026-07-31

**Authors:** Ying Guo, Xiaoxi Niu, Yanlai Xia, Yaqi Yang, Xiuwei Fu, Tongtong Li, Yu Zhang, Yong Ji, Hongyan Ni

**Affiliations:** 1 Department of Radiology, Zhongshan Hospital, Xiamen University, Xiamen, China; 2 Department of Radiology, Tianjin First Central Hospital, Tianjin, China; 3 Department of Radiology, The First Affiliated Hospital of Wenzhou Medical University, Wenzhou, China; 4 Department of Neurology, Tianjin Beichen Hospital, Tianjin, China; 5 Department of Radiology, Tianjin Medical University General Hospital, Tianjin, China; 6 Department of Radiology, The Affiliated Hospital of Hebei University, Baoding, China; 7 Department of Radiology, Cancer Hospital Chinese Academy of Medical Sciences, Beijing, China; 8 Department of Neurology, Tianjin Huanhu Hospital, Tianjin, China

**Keywords:** Alzheimer's disease, dementia with lewy bodies, diffusion MRI, NODDI (neurite orientation dispersion and density imaging), white matter

## Abstract

**Background:**

Dementia with Lewy bodies (DLB) and Alzheimer’s disease (AD) are common types of dementia, however, diagnosis is challenging. An innovative MR method called neurite orientation dispersion and density imaging (NODDI) is used to assess different microstructural alterations between the conditions.

**Objective:**

To assess microstructural white matter (WM) changes in DLB and AD using NODDI.

**Methods:**

Diffusion images were acquired from 26 DLB patients, 36 AD patients, and 37 normal controls (NCs). The diffusion tensor imaging (DTI) was used to generate fractional anisotropy (FA), mean diffusivity (MD), and NODDI was used to generate neurite density index (NDI), orientation dispersion index (ODI), and volume fraction of isotropic water molecules (Viso), these parameters were compared among groups applying Tract-based spatial statistics (TBSS). We also analyzed the correlations between altered parameters and cognitive scores and investigated the diagnostic efficacy of different parameters using k-nearest neighbor (KNN).

**Results:**

Compared with NC, FA, ODI, and NDI significantly decreased, while MD and Viso significantly increased in both DLB and AD. Compared with DLB, ODI and NDI significantly decreased in AD. The diffusion parameters of multiple fibers were strongly correlated with cognition. NDI exhibited a relatively larger AUC value in differentiating between groups.

**Conclusion:**

Widespread disruption of white matter microstructure is evident in both DLB and AD, with differences in these alterations between the two conditions. NDI holds promise as a neuroimaging biomarker for distinguishing DLB from AD.

## Introduction

Dementia with Lewy bodies (DLB) ranks as the second most prevalent neurodegenerative cause of dementia, after Alzheimer's disease (AD). The primary clinical manifestations encompass fluctuating cognitive decline, recurring visual hallucinations, and parkinsonian symptoms such as rigidity, bradykinesia, resting tremor, and postural instability (Peraza et al., 2015). On the other hand, AD is a slowly-onset neurodegenerative disorder and is the most prevalent form of dementia (Kumar et al., 2024). It primarily manifests in cognitive and memory decline and progressive impairment in daily activities (Cao et al., 2020). However, not all DLB patients exhibit early-onset core features, and the clinical manifestations of DLB and AD overlap to some extent. Moreover, DLB lacks of distinctive features on structural MRI. Consequently, the diagnostic accuracy of DLB remains relatively low, posing a challenge in clinical diagnosis for distinguishing DLB from AD. Hence, effectively distinguishing DLB from AD, increasing the diagnosis rate of DLB, and reducing misdiagnosis are the urgent clinical issues that need to be addressed at present.

Some prior studies have suggested that white matter integrity may be compromised in DLB patients (Nicastro et al., 2020; Schumacher et al., 2022). Additionally, certain research studies have proposed that AD is associated with altered white matter microstructure (Yuan et al., 2016; Li et al., 2022). Nonetheless, the patterns of white matter alterations in DLB and AD, along with their potential role in differential diagnosis, remain contentious.

Watson R et al. reported significant differences in diffusion tensor imaging (DTI) patterns between DLB and AD. In contrast, Donaghy PC et al. found no differences between the DLB and AD groups (Watson et al., 2012; Donaghy et al., 2020). Previous DTI studies have yielded conflicting results, suggesting that DTI parameters alone may not provide sufficient sensitivity to characterize WM microstructure changes.

DTI is rooted in the diffusion of water molecules, assuming a Gaussian distribution within a single microstructural compartment. Nevertheless, the water in biological tissue experiences constraints due to interactions with other cell membranes and molecules. Therefore, the proposed hypothesis might not precisely depict the actual dispersion process within biological tissues, underscoring the need for a non-Gaussian model incorporating multiple compartments (Tuch et al., 2003; Jones and Cercignani, 2010). In this investigation, we overcome the limitations of DTI by employing a more advanced multi compartment diffusion model called Neurite Orientation Dispersion and Density Imaging (NODDI). NODDI utilizes a biophysical model that categorizes the diffusion of water molecules in brain tissue into three distinct microstructural environments: intra-neurite, extra-neurite, and cerebrospinal fluid (CSF) compartments. NODDI evaluates parameters such as neurite density index (NDI), orientation dispersion index (ODI), and volume fraction of isotropic water molecules (Viso). NDI quantifies the volume fraction of neurites within intra-neurite spaces, ODI characterizes changes in neurite angles within extra-neurite spaces, reflecting the orientation of fiber distribution in white matter, and Viso represents the volume fraction of water molecules used to describe free diffusion within a voxel, resembling CSF (Zhang et al., 2012; Merluzzi et al., 2016). Mak Elijah et al. reported that DLB exhibits increased MD and FWF, as well as decreased NDI in multiple regions, with focal reductions in ODI in the occipital cortex (Mak et al., 2025).

Previous research (Weston et al., 2023; Zhong et al., 2023) exploring DLB and AD white matter primarily relied on region of interest (ROI) and voxel-based analysis (VBA). However, ROI analysis carries subjectivity and is confined to local areas. VBA statistical results can be influenced by smoothing kernels and cluster sizes. Tract-based Spatial Statistics (TBSS) is a voxel-based whole-brain analysis method that relies on data from all kinds of diffusion models (Smith et al., 2006). The use of TBSS can mitigate the limitations of ROI and VBA-based data analyses. Nevertheless, TBSS is infrequently applied to DLB at present.

In this study, we aim to 1) analyze the pattern of white matter microstructural alterations as detected by NODDI and determine their relationship with ability of cognition in DLB and AD, and 2) to investigate the potential ability of NODDI-derived parameters in distinguishing between DLB and AD.

## Materials and methods

### Subjects

This research received approval from the hospital ethics board, and all participants provided written informed consent before their participation. We enrolled a total of 26 DLB patients, 36 disease severity-matched (assessed with CDR) AD patients, and 37 age and sex matched normal controls in our hospital.

Each subject underwent cognitive evaluations through neuropsychological assessments, which comprised the Mini-Mental State Examination (MMSE), Montreal Cognitive Assessment (MoCA), and Clinical Dementia Rating (CDR) Scales. Furthermore, to exclude specific patients, assessments of the Hachinski Ischemic Scale (HIS) and the Hamilton Depression Rating Scale (HAMD) were also conducted.

Probable DLB was diagnosed based on the diagnostic criteria defined by McKeith in 2017 (McKeith et al., 2017). Patients with Parkinson's disease associated with dementia, who exhibited extrapyramidal syndrome preceding the diagnosis of cognitive impairment by more than one year, were excluded from the study. Probable Alzheimer's disease (AD) diagnoses were made in accordance with the criteria established by the National Institute on Aging and Alzheimer's Association (NIA-AA) (McKhann et al., 2011). The exclusion criteria comprised: (1) patients with contraindications to MRI examination; (2) a history of neuropsychiatric disorders or severe traumatic brain injury; (3) patients with a history of medication affecting cognitive function; (4) individuals with severe diabetes and coronary heart disease; (5) left-handedness; (6) exclusion of severe cerebrovascular disease based on an HIS score ≥ 4; and (7) exclusion of depression based on an HAMD score >7.

Ethical approval for the study was obtained from Tianjin first hospital ethic committee, and written informed consent was obtained from all participants (IRB number: 2018N141KY).

### MRI acquisition

All subjects were scanned using a 3T MAGNETOM Prisma MR scanner (Siemens Healthcare, Erlangen, Germany) equipped with a 64-channel head-neck coil. The diffusion images were acquired using the spin-echo echo-planar imaging (SE-EPI) sequence with the following specific parameters: TR/TE = 3800/72 ms, FOV = 220 mm × 220 mm, voxel size = 2.0 mm × 2.0 mm × 2.2 mm, matrix = 110 × 110, 60 slices, 64 diffusion directions, b-values of 0/2000/3000 s/mm^2^, simultaneous multi-slice acquisition (SMS) acceleration factor = 2, and total scan time= 8 min and 30 s. For the MPRAGE sequence, the parameters were as follows: TR/TE = 1550/2.98 ms, 176 slices, voxel size = 1.0 mm × 1.0 mm × 1.0 mm, FOV = 256 mm × 256 mm, and flip angle = 9°.

### MRI data processing

Processing of diffusion MRI data involved the following steps: 1) all raw diffusion images were converted from Digital Imaging and Communications in Medicine (DICOM) format to the Neuroimaging Informatics Technology Initiative (NIFTI) format, 2) utilization of the FSL toolbox (Smith et al., 2004). The correction of head motion-related artifacts and the addressing of image distortion caused by eddy currents were carried out using the tools provided by the Oxford Centre for Functional MRI of the Brain.^1^ Subsequently, the calculation of Diffusion Tensor Imaging (DTI) parameters, specifically Fractional Anisotropy (FA) and Mean Diffusivity (MD), was performed using the DTIFIT tools within FSL. Brain Extraction Tool (BET) in FSL was applied to extract non-brain tissue, then we utilized the NODDI_toolbox^2^ to obtained a brain mask in Matlab (Mathworks, Natick, MA, United States), and the brain mask was acquired to define the NODDI processing regions. The NODDI parameters, such as NDI, ODI, and Viso, were computed using the WatsonSHStickTortIsoV_B0 model (Zhang et al., 2012). We applied the TBSS method to all parameters for statistical analysis.

### Tract-based spatial statistics analysis

To examine microstructural changes in white matter, we employed the Tract-Based Spatial Statistics (TBSS) method following the standardized TBSS framework. Initially, all participant images were nonlinearly registered to the FMRIB58_FA template in the Montreal Neurological Institute (MNI) standard space. Subsequently, we computed the average of all aligned images in the MNI space, creating a mean Fractional Anisotropy (FA) skeleton with a threshold value of 0.2. The aligned FA maps were then projected onto this mean FA skeleton. Additionally, non-FA maps (including MD, Viso, NDI, and ODI) were also projected onto the same mean FA skeleton using the identical non-linear registration. Finally, voxel-wise statistical analyses were conducted across participants on this common skeleton.

For TBSS analyses, we implemented a general linear model with a one-way ANOVA in FSL, incorporating sex and age as covariates. To examine group differences, non-parametric permutation tests were conducted with 5000 permutations using randomization commands. Multiple comparison corrections were applied using Threshold-Free Cluster Enhancement (TFCE), and statistical significance was defined as *p* < 0.05. TBSS results indicating *p* < 0.05 were saved as a mask in FSL. A post-hoc two-sample test was then carried out between any two groups within the mask. Additionally, the TBSS results mask was overlaid on Johns Hopkins University JHU-ICBM-labelled templates (48 white matter tracts) in the MNI standard space. This overlay was saved as a new mask using SPM software in Matlab2016b. Finally, with the newly saved mask, we utilized the DPABI software for further analysis^3^ package to extract parameter values in regions with significant differences between groups.

### Statistical analyses

Demographic data and neuropsychological scores of all participants were subjected to statistical analysis using SPSS 26.0 software (IBM Corp., Armonk, NY, United States). Normal distribution and homogeneity of variance were assessed for all quantitative data. The Shapiro-Wilk test was employed for normality testing, while Levene's test was utilized for assessing homogeneity of variance. The age followed a normal distribution, while the MMSE, MoCA, CDR scores and years of education did not. The diffusion parameters (kurtosis < 10 and skewness < 3) are generally acceptable as approximating normality. For quantitative data exhibiting a normal distribution, one-way ANOVA was applied, and non-parametric tests were used for data lacking normal distributions. Sex analysis among the three groups was conducted using the *χ*^2^-test. A significance level of *p* < 0.05 was considered indicative of statistical significance. Diffusion parameters with significant differences between the DLB vs NC group and AD vs NC group were identified, and the correlation between image parameter values and cognitive scores was analyzed using Spearman’s correlation, adjusting for age, sex and education years.

We used K-nearest neighbor (KNN) classifier with the scikit-learn library in Python to establish classification model and investigate the diagnostic power of diffusion metrics for DLB and AD. To optimize model performance, 5-fold stratified cross-validation was applied to determine the optimal hyperparameters, including the number of neighbors (k), weighting methods and distance metrics. Feature standardization and grid search for optimal k were performed exclusively within each training fold to prevent data leakage, and the optimal k for each classifier was summarized in a table (Supplementary Table S4). We further examined sex distribution within each fold of stratified five-fold cross-validation. All out-of-fold predicted probabilities across five validation subsets were pooled to form a single set of predicted values matched to the true binary labels of all participants. A single overall cross-validated AUC was calculated based on this pooled dataset, and its 95% confidence interval was estimated using the DeLong method to quantify uncertainty.

## Results

### Demographics

Table 1 presents the demographic data and neuropsychological scores. No significant differences were observed in sex (*p* = 0.254), age (*p* = 0.120), and education years (*p* = 0.102) among the DLB, AD, and NC groups. However, significant differences were noted in MMSE (*p* < 0.001), MoCA (*p* < 0.001), and CDR scores (*p* < 0.001) between the DLB and NC groups, as well as between the AD and NC groups. Importantly, patients with DLB did not show significant differences from those with AD in MMSE (*p* = 0.656), MoCA (*p* = 0.851), or CDR scores (*p* = 0.793).

**Table 1:** Demographic data and neuropsychological scores analyses.

Parameters	DLB (*n* =26)	AD (*n* = 36)	NC (*n* = 37)	*p*-value
Sex (M/F)	14/12	12/24	17/20	0.254
Age (years)	73.54 ±7.79	71.61 ±9.00	69.46 ±6.23	0.120
Education(years)	12.0 (9.0 ~15.0)	9.5 (9.0 ~12.0)	12.0 (9.0 ~16.0)	0.102
MMSE	15.0 (9.8~20.0)^a^	14.0 (8.0~19.0^a^	29.0 (28.0~30.0)	<0.001
MoCA	10.5 (6.0~15.3)^a^	9.0 (7.0~14.0)^a^	27.0 (26.0~29.0)	<0.001
CDR	1.0(0.5~2.0)^a^	1.0 (1.0~2.0)^a^	0.0 (0.0~0.0)	<0.001
Fluctuating cognition, *n* (%)	10 (38.5)	/	/	/
Visual hallucinations, *n* (%)	13 (50.0)	/	/	/
REM sleep behavior disorder, *n* (%)	15 (57.7)	/	/	/
Parkinsonism, *n* (%)	18 (69.2)	/	/	/

A *χ*^2^-test was performed for sex comparison.

A one-way ANOVA was used to compare age.

A Kruskal–Wallis test was used to compare years of education, MMSE, MoCA and CDR scores, and the results are presented as median (lower quartile ~ upper quartile).

MMSE, mini-mental state examination; MoCA, Montreal cognitive assessment; CDR, Clinical Dementia Rating; AD, Alzheimer’s disease; DLB, dementia with Lewy bodies; NC, normal control; M, male; F, female; ANOVA, analysis of variance; a, Significantly different compared with the NC group.

### TBSS analyses

**Figure 1:**
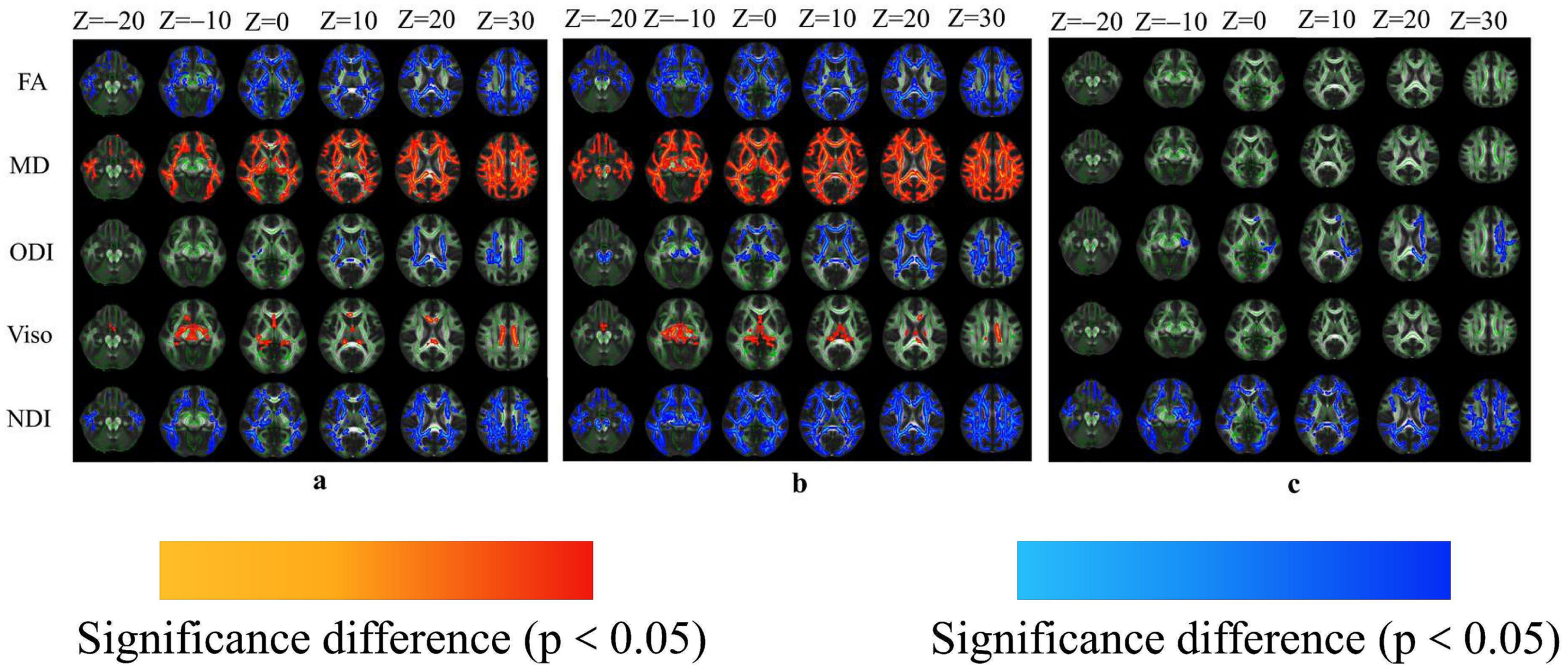
**(a–c)** The results of comparisons between DLB and NC, AD and NC, and DLB and AD, respectively. The green color represents the skeleton. Blue signifies brain structures with significantly decreased parameter values in both DLB and AD groups (*p* < 0.05, TFCE-corrected), while red-yellow represents brain structures with significantly increased parameter values in both DLB and AD groups (*p* < 0.05, TFCE-corrected).

Compared to the NC group, individuals with DLB demonstrated noteworthy decreases in FA, ODI, and NDI values (*p* < 0.05, TFCE-corrected) (Supplementary Table S1). Regions of the brain where FA values significantly decreased included the corpus callosum, fornix, bilateral internal capsule, left corona radiata, left posterior thalamic radiation, left sagittal stratum, and bilateral superior longitudinal fasciculus. Additionally, significant reductions in ODI values were observed in the bilateral internal capsule, bilateral corona radiata, right posterior thalamic radiation, and the right superior longitudinal fasciculus. Similarly, significant reductions in NDI values were observed in key brain areas, including bilateral internal capsule, bilateral corona radiata, right posterior thalamic radiation, right sagittal stratum, left external capsule, and bilateral superior longitudinal fasciculus. Conversely, Mean Diffusivity (MD) and Volume Fraction of Isotropic Water Molecules (Viso) values exhibited notable increases in DLB patients (*p* < 0.05, TFCE-corrected) (Supplementary Table S1). Specifically, elevated MD values were detected in the corpus callosum, bilateral internal capsule, bilateral corona radiata, bilateral posterior thalamic radiation, bilateral sagittal stratum, left external capsule, right cingulum, and bilateral superior longitudinal fasciculus. Likewise, increased Viso values were observed in the corpus callosum, fornix, and the right retrolenticular part of the internal capsule (Figure 1a).

In contrast to the NC group, individuals with AD exhibited significant reductions in Fractional Anisotropy (FA), Orientation Dispersion Index (ODI), and Neurite Density Index (NDI) values (*p* < 0.05, TFCE-corrected) (Supplementary Table S2). Specifically, brain regions with significantly reduced FA values encompassed the middle cerebellar peduncle, fornix, right corticospinal tract, bilateral internal capsule, bilateral corona radiata, left posterior thalamic radiation and bilateral sagittal stratum. Areas with significantly decreased ODI values included bilateral internal capsule, right corona radiata, right posterior thalamic radiation, bilateral external capsule, bilateral cingulum, and bilateral superior longitudinal fasciculus. Additionally, regions with significantly reduced NDI values included the middle cerebellar peduncle, corpus callosum, fornix, right corticospinal tract, bilateral cerebral peduncle, bilateral internal capsule, bilateral corona radiata, left posterior thalamic radiation, left sagittal stratum, bilateral external capsule, right cingulum and left superior longitudinal fasciculus However, Mean Diffusivity (MD) and Volume Fraction of Isotropic Water Molecules (Viso) values exhibited a significant increase in AD patients (*p* < 0.05, TFCE-corrected) (Supplementary Table S2). Notably, brain regions with notably increased MD values included corpus callosum, bilateral internal capsule, bilateral corona radiata, bilateral posterior thalamic radiation, right sagittal stratum, left external capsule, left cingulum, and bilateral superior longitudinal fasciculus. Additionally, areas with significantly increased Viso values included the body of the corpus callosum, splenium of the corpus callosum, fornix, right cerebral peduncle, and bilateral posterior limb of the internal capsule (Figure 1b).

When comparing AD patients with the DLB group, Orientation Dispersion Index (ODI) and Neurite Density Index (NDI) values were significantly decreased in AD patients (*p* < 0.05, TFCE-corrected) (Supplementary Table S3). Brain regions with significantly reduced ODI values included the corpus callosum, fornix, left internal capsule, left corona radiata, left posterior thalamic radiation, left sagittal stratum, left external capsule, and left superior longitudinal fasciculus. Regions with significantly reduced NDI values comprised the corpus callosum, left cerebral peduncle, bilateral internal capsule, bilateral corona radiata, right posterior thalamic radiation, right sagittal stratum, bilateral external capsule, left cingulum, and bilateral superior longitudinal fasciculus. However, there were no significant differences in Fractional Anisotropy (FA), Mean Diffusivity (MD), and Volume Fraction of Isotropic Water Molecules (Viso) values between the DLB and AD groups (*p* > 0.05, TFCE-corrected) (Figure 1c).

### Correlations between imaging parameters and neuropsychological scores

**Figure 2:**
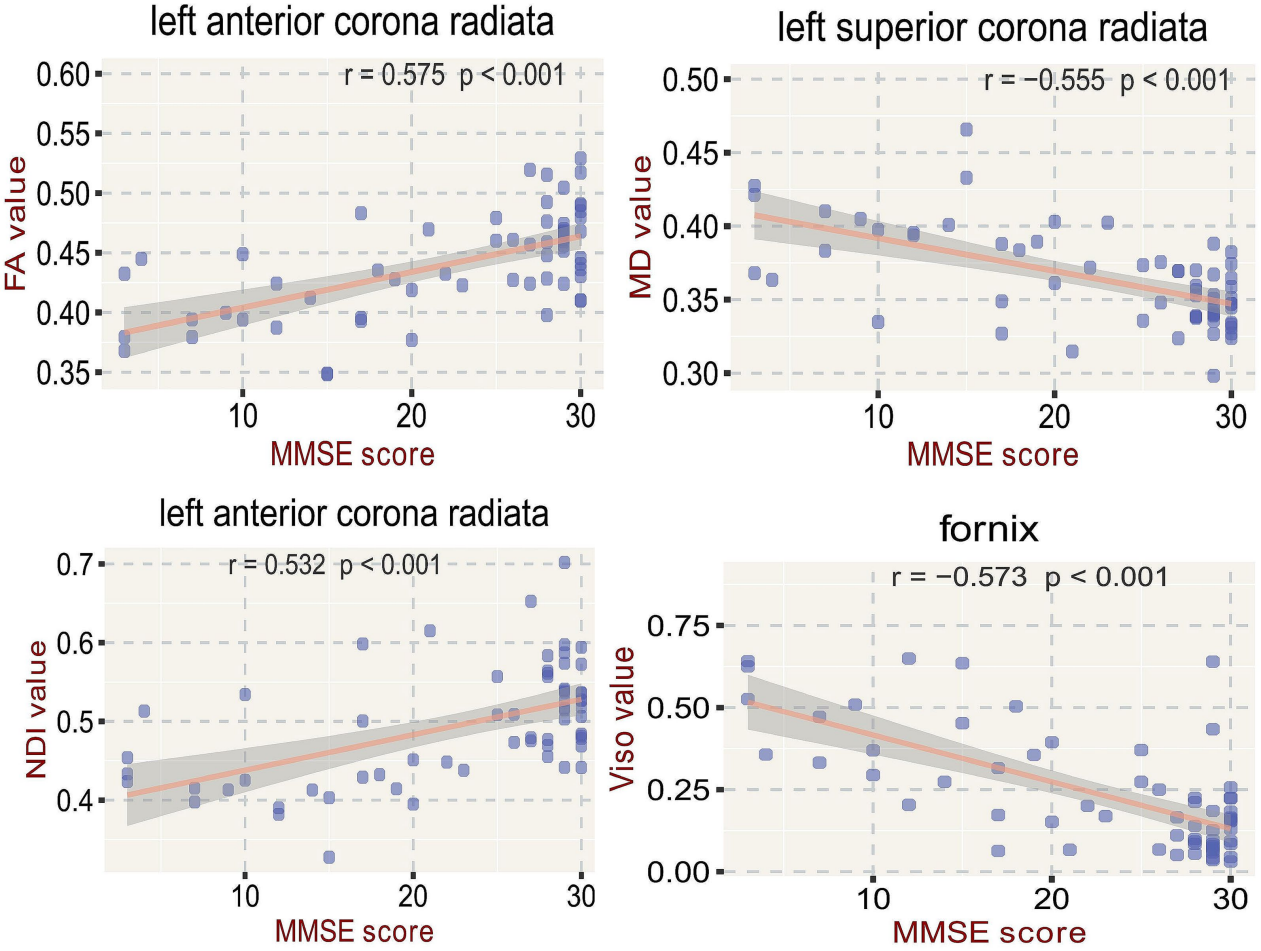
Presents the correlation analyses, showing correlation coefficients between DTI-derived and NODDI-derived parameters (namely FA, MD (×10^-3^ mm^2^/s), NDI, and Viso), and the neuropsychological testing scores (specifically MMSE scores) within the DLB group.

**Figure 3:**
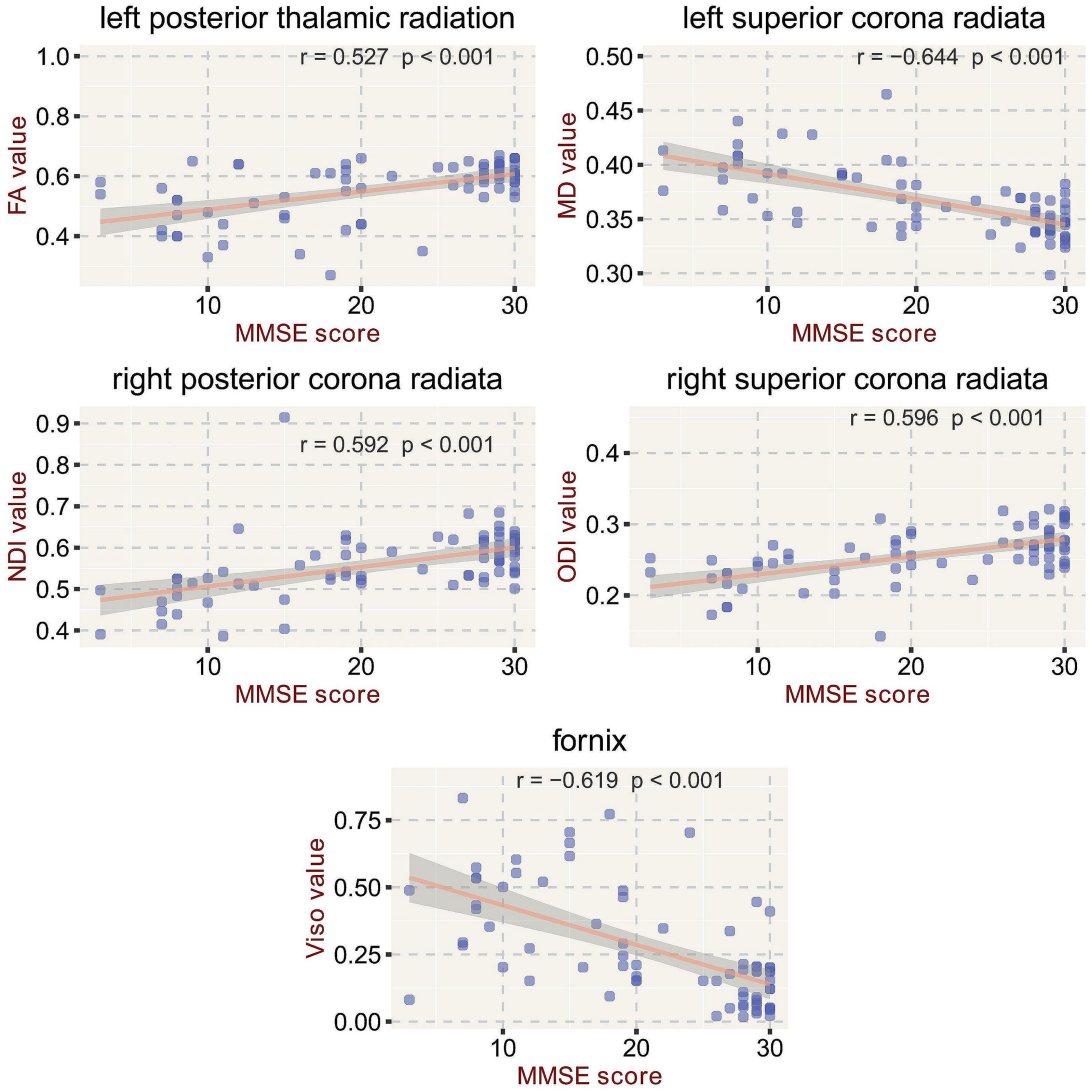
The correlation analyses, illustrating the correlation coefficients between DTI-derived and NODDI-derived parameters (FA, MD (×10^-^3 mm2/s), NDI, ODI, and Viso) and the neuropsychological testing scores (MMSE scores) in AD group.

The TBSS results revealed notable correlations between the parameter values of white matter tracts, which exhibited statistical differences between groups, and MMSE scores. In both DLB and AD groups, Fractional Anisotropy (FA), Orientation Dispersion Index (ODI), and Neurite Density Index (NDI) values in most fibers demonstrated significant positive correlations with MMSE scores. Conversely, Mean Diffusivity (MD) and Volume Fraction of Isotropic Water Molecules (Viso) values exhibited significant negative correlations with MMSE scores. Detailed correlation graphs are presented in Figures 2, 3.

Within the DLB group, strong positive correlations were observed between Fractional Anisotropy (FA) and Neurite Density Index (NDI) values of the left anterior corona radiata and Mini-Mental State Examination (MMSE) scores (*r* = 0.575, *p* < 0.001; *r* = 0.532, *p* < 0.001). Conversely, strong negative correlations were noted between Mean Diffusivity (MD) values of the left superior corona radiate (*r* = –0.555, *p* < 0.001) and Volume Fraction of Isotropic Water Molecules (Viso) values of the fornix (*r* = –0.573, *p* < 0.001) with MMSE scores.

Within the AD group, strong positive correlations were evident between Fractional Anisotropy (FA) values of the left posterior thalamic radiation (*r* = 0.527, *p* < 0.001), Orientation Dispersion Index (ODI) values of the right superior corona radiata (*r* = 0.596, *p* < 0.001), and Neurite Density Index (NDI) values of the right posterior corona radiata (*r* = 0.592, *p* < 0.001) with Mini-Mental State Examination (MMSE) scores. Conversely, strong negative correlations were observed between Mean Diffusivity (MD) values of the left superior corona radiata (*r* = –0.644, *p* < 0.001) and Volume Fraction of Isotropic Water Molecules (Viso) values of the fornix (*r* = –0.619, *p* < 0.001) and MMSE scores.

### KNN analyses for diagnostic performance

KNN with 5-fold cross-validation analyses were conducted using different FA, MD, ODI, Viso, and NDI parameters to assess diagnostic performance. For discriminating the AD group from the DLB group, the NDI exhibited numerically slightly higher AUC values (AUC=0.760, DeLong 95% CI: 0.597-0.924) than the ODI value (AUC = 0.508, DeLong 95% CI: 0.310-0.706). In differentiating the DLB group from the NC group, NDI produced a relatively larger AUC value (AUC = 0.735, DeLong 95% CI: 0.575-0.896), while ODI showed a comparatively smaller AUC estimate (AUC = 0.511, DeLong 95% CI: 0.314-0.707). Finally, for distinguishing the AD group from the NC group, NDI had a marginally higher numerical AUC (AUC = 0.854, DeLong 95% CI: 0.762-0.946), while Viso yielded a relatively lower AUC value (AUC = 0.661, DeLong 95% CI: 0.520-0.803) (Table 2).

**Table 2:** Classification performance of diffusion indexes using 5-fold cross-validation.

Group	Parameters	AUC	DeLong 95% confidence interval
DLB<AD	NDI	**0.760**	[0.597, 0.924]
	ODI	0.508	[0.310, 0.706]
NC<DLB	FA	0.606	[0.437, 0.775]
	MD	0.707	[0.535, 0.879]
	NDI	**0.735**	[0.575, 0.896]
	ODI	0.511	[0.314, 0.707]
	VISO	0.629	[0.459, 0.799]
NC<AD	FA	0.712	[0.587, 0.836]
	MD	0.809	[0.685, 0.932]
	NDI	**0.854**	[0.762, 0.946]
	ODI	0.823	[0.716, 0.929]
	VISO	0.661	[0.520, 0.803]

Bold number indicate the highest AUC for each group of parameters.

## Discussion

In this investigation, we employed FA, MD, ODI, Viso, and NDI to assess alterations in white matter microstructure. Simultaneously, we explored the capability of NODDI in detecting microstructural changes in white matter for individuals with DLB and AD, comparing it with conventional DTI. The key findings were as follows: (1) In comparison to the NC group, there were significant decreases in FA, ODI, and NDI values, accompanied by significant increases in MD and Viso values in both DLB and AD patients, indicating abnormalities in brain microstructure for both conditions; (2) When contrasting AD with DLB patients, significant decreases were observed in ODI and NDI values, while no significant differences were found in FA and MD values, suggesting that NODDI might offer advantages over DTI in distinguishing between DLB and AD; (3) Various diffusion parameter values in several fiber pathways, including the left posterior thalamic radiation, fornix, and left anterior corona radiata, were significantly correlated with MMSE scores, indicating microstructural abnormalities associated with cognitive decline; (4) NDI may serve as a new imaging marker for the differentiation between AD and DLB.

We observed significant alterations in diffusion parameters between NC and patients with DLB and AD. Specifically, we found a significant decrease in FA, ODI, and NDI values, accompanied by a significant increase in MD and Viso values in both DLB and AD patients. These findings indicate pronounced microstructural abnormalities in the brains of individuals with DLB and AD, which are consistent with previous research (Nicastro et al., 2020; Li et al., 2022; Xiao et al., 2022). Furthermore, when comparing AD patients with NC, we observed a decrease in ODI and NDI values, signifying a reduction in white matter integrity. These findings align with previous histopathological studies that have demonstrated axonal degeneration and myelin abnormalities in AD (Baloyannis et al., 2011). In addition, it has been shown that myelin renewal improves cognitive dysfunction in AD mice (Lacalle-Aurioles and Iturria-Medina, 2023). Studies have also suggested that hyperphosphorylation of Tau protein may lead to synaptic loss and dendritic spine shortening in AD (Mitsuyama et al., 2009), contributing to the reduction of NDI. A previous study in a mouse model also found that NDI values correlated with hyperphosphorylated Tau levels (Sone et al., 2020). Neurodegenerative changes lead to reduced dendritic branching and loss of dendritic spines, resulting in reduced dendrite complexity (Boros et al., 2017), which could be the reason for the lower ODI values. The reason for the increase in Viso values may be attributed to disruptions in the integrity of the myelin sheath (Wang et al., 2018) and the presence of brain atrophy, which can increase cerebrospinal fluid around the cerebral cortex (Colgan et al., 2015). Similarly, DLB patients exhibited significant alterations in white matter microstructure, consistent with the notion that changes in white matter structure are an early feature of DLB (Firbank et al., 2016). It has been suggested that DLB patients experience neuron loss and axonal degeneration, which could explain the changes in NODDI parameters compared to NC (Smith et al., 2019). Specifically, changes in diffusion parameters in the DLB group overlapped in the corpus callosum, the posterior thalamic radiation, and the superior longitudinal fasciculus. The corpus callosum is the largest white matter fiber tract connecting the left and right cerebral hemispheres, and the integrity of its microstructure is crucial for efficient interhemispheric information transfer. Nicastro et al. (2020) had confirmed that microstructural damage to the corpus callosum may mediate cognitive decline in DLB patients, negatively affecting functions such as executive control and attention. The posterior thalamic radiations are key components of the visual conduction pathway, primarily responsible for transmitting visual information from the lateral geniculate nucleus to the primary visual cortex in the occipital lobe. A previous study (Laurell et al., 2025) found that DLB patients exhibit more severe damage in thalamic connections and the substantia nigra–striatum pathway, directly supporting the notion of impaired posterior thalamic radiations in DLB. Microstructural damage to the superior longitudinal fasciculus is closely related to language and memory abilities. As a major pathway connecting widespread brain regions, its impairment is likely one of the neural bases underlying the complex clinical symptoms of DLB, such as cognitive fluctuations, attentional deficits, executive dysfunction, and language impairment. One study (Zorzi et al., 2021) on DLB patients indicated that the degree of microstructural abnormalities in the right superior longitudinal fasciculus was positively correlated with the severity of visual hallucinations. Additionally, damage to the superior longitudinal fasciculus was negatively associated with performance on visual attention tasks, which helps explain the commonly observed attentional fluctuations in DLB patients.

Importantly, we compared diffusion parameters between DLB and AD patients in our study. We found no significant differences in FA and MD values, consistent with prior research (Firbank et al., 2011). Additionally, AD exhibited significantly decreased NDI and ODI in the corpus callosum, corona radiata, and posterior thalamic radiation. These regions are critical for interhemispheric communication, sensorimotor integration, and thalamo-cortical relay, respectively. The more severe microstructural impairment in these major white matter tracts may underlie the more prominent global cognitive decline and widespread functional deficits observed in AD (Lv et al., 2025). This result aligns with Watson R et al.’s findings, which similarly reported more widespread microstructure changes in AD compared to DLB patients (Watson et al., 2012), supporting our results. Fu et al. also demonstrated the effectiveness of NODDI in assessing white matter microstructure by showing no significant FA differences but significant ODI and NDI changes, along with increased Viso values in mild cognitive impairment patients compared to the NC (Fu et al., 2020). Most previous studies on white matter microstructure in DLB used DTI, which has lower specificity due to its susceptibility to axon diameter, density, fiber distribution direction, and partial volume effects. NODDI, on the other hand, can assess neurite density and fiber distribution direction separately, providing a more accurate assessment of tissue microstructure changes and advantages in diagnosing neurodegenerative diseases (Andica et al., 2020; Broad et al., 2018). Compared to DTI, NODDI can sensitively assess neurite density and fiber dispersion, offering greater specificity for microstructural changes and insights into the structural basis of brain function in normal individuals and those with brain diseases (Zhang et al., 2012). The differences we found in white matter microstructure between DLB and AD in this study may aid in differential diagnosis.

Robust correlations between DTI parameters and neuropsychological scores were identified in numerous regions, especially concerning the FA values of the left anterior corona radiata in DLB patients and the MD values of the left superior corona radiata in AD patients. This substantiates the connection between cognition and alterations in white matter microstructure. The evidence suggests that the corona radiata is a pivotal projection fiber, contributing significantly to memory, language-related processing, and various cognitive functions (Olivo et al., 2017). Regarding the correlations between NODDI parameters and neuropsychological scores, the Viso values within the fornix displayed the most pronounced associations with MMSE scores. This finding aligns with the results of Lacalle-Aurioles et al. (Lacalle-Aurioles and Iturria-Medina, 2023). The fornix, originating from the medial portion of the hippocampus, emerges as a crucial structure in learning and memory processes. Furthermore, the study reported a correlation between cerebrospinal fluid amyloid-β (Aβ) and elevated Viso values, as observed by Reas et al. (2020). This underscores that Viso values not only partly reflect the pathological deposition in dementia patients but also offer insights into disease severity. Among the 36 AD patients we studied, there were 25 patients with mild to moderate AD and 11 patients with severe AD(MMSE<10). Among the patients with severe AD, the MMSE scores of two of the patients were extremely low (MMSE = 3), and the patients lose communicative abilities, become fully dependent on caregivers for all activities of daily living, and develop motor rigidity and swallowing difficulties. The profound cognitive and functional impairment in our sample explains the floor effects observed on the MMSE, and represents a specific population of advanced AD that is under-represented in many clinical studies.

In our analysis of the diagnostic performance of diffusion parameters using the KNN model, We observed that, compared with other diffusion metrics, NDI tended to show relatively greater diagnostic capacity for intergroup discrimination across the three groups. The result suggested that NDI enables more precise detection of microstructural white matter differences between AD and DLB, as a marker of axonal density and white matter tract complexity. Consistent with a previous study (Fu et al., 2020), we also found that NODDI might have advantages over DTI in the diagnosis of AD and DLB (Lv et al., 2025).

It is crucial to acknowledge the limitations of our study. Firstly, the acquisition of image data was challenging due to the limited cooperation of DLB patients. The sample size of DLB group (*n* = 26) was relatively small, this may restrict the generalizability of our findings. Thus, future studies with larger and more diverse cohorts are needed to validate the results. In addition, NDI is interpreted as neurite density, NODDI parameters are model-derived estimates rather than direct histological measurements—particularly in aged or diseased brains where gliosis, edema, or vascular alterations may violate the assumptions of the NODDI model. In future studies, we will further incorporate the free water (FW) parameter for correction. Moreover, the study did not include hallmark pathological features (Aβ plaques and tau tangles) due to these biomarkers can be assessed using PET imaging or cerebrospinal fluid (CSF) analysis, these methods are invasive, expensive, or not widely accessible, making them less ideal for routine screening, which limits their clinical feasibility and may lead to potential misclassification. In future research, we will gradually incorporate these markers to further enhance the robustness of our findings. Finally, our study design was cross-sectional, which limits our capacity to establish causal relationships or explore the temporal dynamics of white matter changes. Longitudinal studies tracking white matter microstructure over time would offer valuable insights into disease progression and potentially shed light on the role of white matter alterations in disease trajectory and cognitive decline.

In conclusion, our study highlights widespread disruptions in white matter microstructure in DLB and AD patients, with notable differences between the two conditions. NODDI appears to be a valuable tool for detecting microstructural changes, and NDI holds promise as a neuroimaging biomarker for the differential diagnosis of DLB and AD.

## Data Availability

The raw data supporting the conclusions of this article will be made available by the authors, without undue reservation.

## Footnotes

1
http://fsl.fmrib.ox.ac.uk/fsl/fslwiki/

2
www.nitrc.org/projects/noddi_toolbox

3
http://rfmri.org/dpabi
