# Supplementary material for: Microstructural white matter alterations in dementia with lewy bodies and Alzheimer’s disease: a diffusion tensor imaging and neurite orientation dispersion and density imaging study
Source: Front Aging Neurosci. 2026 Jul 31;18:1830477. doi: 10.3389/fnagi.2026.1830477 (PMC13472807; doi:10.3389/fnagi.2026.1830477)
Supplement: Supplementary file 1 [file Supplementary_File_1.pdf]

Supplementary Material

Microstructural White Matter Alterations in Dementia with Lewy Bodies and Alzheimer's Disease: A Diffusion Tensor Imaging (DTI) and Neurite Orientation Dispersion and Density Imaging (NODDI) Study

Ying Guo[1†], Xiaoxi Niu[2†], Yanlai Xia[3], Yaqi Yang[4], Xiuwei Fu[5], Tongtong Li[6], Yu Zhang[7], Yong Ji[8], Hongyan Ni[9*]
1Department of Radiology, Zhongshan Hospital, Xiamen University, Xiamen, China
2Department of Radiology, Tianjin First Central Hospital, Tianjin, China
3Department of Radiology, the First Affiliated Hospital of Wenzhou Medical University, Wenzhou, China
4Department of Neurology, Tianjin Beichen Hospital, Tianjin, China
5Department of Radiology, Tianjin Medical University General Hospital, Tianjin, China
6Department of Radiology, The Affiliated Hospital of Hebei University, Baoding, China
7Department of Radiology, Cancer Hospital Chinese Academy of Medical Sciences, Beijing, China
8Department of Neurology, Tianjin Huanhu Hospital, Tianjin, China
9Department of Radiology, Tianjin First Central Hospital, Tianjin, China
†These authors have contributed equally to this work and share first authorship
***CORRESPONDENCE**
Hongyan Ni
hongyni@163.com

Table 1. A comparison of diffusion parameters in the white matter of the DLB patients vs. the NC subjects

	ROI	DLB	NC	Cohen's d
FA	corpus callosum	0.580±0.090	0.630±0.050	-0.722
	fornix	0.410±0.110	0.450±0.100	-0.384
	left internal capsule	0.630±0.050	0.670±0.050	-0.800
	right internal capsule	0.580±0.070	0.600±0.080	-0.263
	left anterior corona radiata	0.420±0.030	0.460±0.030	-1.333
	left posterior thalamic radiation	0.540±0.080	0.610±0.040	-1.172
	left sagittal stratum	0.450±0.060	0.490±0.060	-0.667
	right superior longitudinal fasciculus	0.453±0.067	0.463±0.043	-0.196
	left superior longitudinal fasciculus	0.540±0.060	0.550±0.060	-0.167
MD ($\times 10^{-3}mm^2/s$)	corpus callosum	0.420±0.030	0.410±0.020	0.407
	right internal capsule	0.350±0.020	0.340±0.010	0.670
	left internal capsule	0.360±0.030	0.340±0.020	0.813
	right anterior corona radiata	0.400±0.030	0.390±0.020	0.407
	left anterior corona radiata	0.420±0.040	0.380±0.020	1.339

	right superior corona radiata	0.350±0.030	0.330±0.010	0.967
	left superior corona radiata	0.380±0.030	0.350±0.020	1.220
	right posterior corona radiata	0.380±0.040	0.350±0.020	1.005
	left posterior corona radiata	0.410±0.030	0.390±0.020	0.813
	right posterior thalamic radiation	0.400±0.030	0.380±0.020	0.813
	left posterior thalamic radiation	0.420±0.020	0.400±0.020	1.000
	right sagittal stratum	0.410±0.030	0.400±0.020	0.407
	left sagittal stratum	0.440±0.030	0.430±0.020	0.407
	left external capsule	0.450±0.030	0.440±0.010	0.483
	right cingulum	0.380±0.030	0.370±0.020	0.407
	right superior longitudinal fasciculus	0.389±0.028	0.372±0.020	0.750
	left superior longitudinal fasciculus	0.360±0.030	0.340±0.010	0.967
ODI	right internal capsule	0.270±0.040	0.280±0.040	-0.250
	left internal capsule	0.200±0.030	0.210±0.040	-0.276
	right anterior corona radiata	0.350±0.030	0.360±0.030	-0.333
	right superior corona radiata	0.260±0.040	0.280±0.030	-0.581
	left superior corona radiata	0.260±0.030	0.270±0.020	-0.407
	right posterior corona radiata	0.260±0.050	0.280±0.030	-0.507
	right posterior thalamic radiation	0.260±0.040	0.280±0.050	-0.433
	right superior longitudinal fasciculus	0.330±0.050	0.340±0.030	-0.254
Viso	genu of the corpus callosum	0.190±0.070	0.120±0.040	1.288
	body of the corpus callosum	0.310±0.150	0.190±0.070	1.090
	splenium of the corpus callosum	0.330±0.160	0.230±0.100	0.781
	right retrolenticular part of the internal capsule	0.100±0.080	0.060±0.020	0.748
	fornix	0.370±0.200	0.140±0.090	1.581
NDI	right internal capsule	0.540±0.070	0.570±0.040	-0.552
	left internal capsule	0.630±0.060	0.660±0.050	-0.552
	right anterior corona radiata	0.500±0.080	0.530±0.050	-0.469
	left anterior corona radiata	0.460±0.070	0.530±0.060	-1.089
	right superior corona radiata	0.630±0.070	0.670±0.040	-0.736
	left superior corona radiata	0.570±0.070	0.630±0.050	-1.017
	right posterior corona radiata	0.550±0.070	0.590±0.050	-0.678
	left posterior corona radiata	0.520±0.080	0.530±0.050	-0.156
	right posterior thalamic radiation	0.520±0.060	0.560±0.040	-0.813
	right sagittal stratum	0.480±0.060	0.500±0.040	-0.407
	left external capsule	0.450±0.050	0.470±0.030	-0.507
	right superior longitudinal fasciculus	0.570±0.070	0.600±0.050	-0.508
	left superior longitudinal fasciculus	0.610±0.080	0.640±0.040	-0.502

Table 2. A comparison of diffusion parameters in the white matter of the AD patients vs. the NC subjects

	ROI	AD	NC	Cohen's d

FA	middle cerebellar peduncle	0.450±0.040	0.47±0.040	-0.500
	genu of the corpus callosum	0.520±0.090	0.550±0.060	-0.393
	body of the corpus callosum	0.570±0.090	0.630±0.050	-0.827
	splenium of the corpus callosum	0.590±0.070	0.660±0.050	-1.153
	fornix	0.400±0.110	0.450±0.100	-0.476
	right corticospinal tract	0.550±0.040	0.560±0.040	-0.250
	right internal capsule	0.600±0.070	0.670±0.050	-1.153
	left internal capsule	0.540±0.060	0.600±0.040	-1.180
	right corona radiata	0.420±0.040	0.460±0.030	-1.134
	left corona radiata	0.490±0.070	0.550±0.020	-1.173
	left posterior thalamic radiation	0.500±0.10	0.610±0.040	-1.452
	right sagittal stratum	0.520±0.050	0.550±0.040	-0.664
	left sagittal stratum	0.440±0.080	0.490±0.060	-0.709
MD ($\times 10^{-3}mm^2/s$)	corpus callosum	0.430±0.030	0.410±0.020	0.787
	right internal capsule	0.400±0.020	0.380±0.020	1.000
	left internal capsule	0.370±0.020	0.340±0.020	1.500
	right anterior corona radiata	0.420±0.030	0.390±0.020	1.180
	left anterior corona radiata	0.420±0.040	0.380±0.020	1.270
	right superior corona radiata	0.360±0.030	0.330±0.010	1.349
	left superior corona radiata	0.380±0.030	0.350±0.020	1.180
	right posterior corona radiata	0.390±0.030	0.350±0.020	1.573
	left posterior corona radiata	0.410±0.040	0.390±0.020	0.635
	right posterior thalamic radiation	0.410±0.030	0.380±0.020	1.180
	left posterior thalamic radiation	0.410±0.030	0.400±0.020	0.393
	right sagittal stratum	0.430±0.030	0.400±0.020	1.180
	left external capsule	0.450±0.020	0.440±0.010	0.635
	left cingulum	0.480±0.020	0.470±0.020	0.500
	right superior longitudinal fasciculus	0.390±0.030	0.370±0.020	0.787
	left superior longitudinal fasciculus	0.370±0.020	0.340±0.010	1.905
ODI	right internal capsule	0.270±0.070	0.280±0.040	-0.176
	left internal capsule	0.180±0.020	0.210±0.040	-0.945
	right anterior corona radiata	0.340±0.030	0.360±0.030	-0.667
	right superior corona radiata	0.240±0.030	0.280±0.030	-1.333
	right posterior corona radiata	0.250±0.060	0.280±0.030	-0.635
	right posterior thalamic radiation	0.240±0.040	0.280±0.050	-0.882
	right sagittal stratum	0.390±0.040	0.400±0.050	-0.221
	left external capsule	0.270±0.020	0.280±0.020	-0.500
	right cingulum	0.280±0.090	0.330±0.070	-0.621
	left cingulum	0.360±0.070	0.390±0.050	-0.494
	right superior longitudinal fasciculus	0.320±0.040	0.340±0.030	-0.567

	left superior longitudinal fasciculus	0.260±0.030	0.280±0.020	-0.787
Viso	body of the corpus callosum	0.340±0.180	0.190±0.070	1.104
	splenium of the corpus callosum	0.380±0.140	0.230±0.100	1.236
	right cerebral peduncle	0.410±0.080	0.340±0.050	1.053
	right posterior limb of the internal capsule	0.090±0.020	0.080±0.020	0.500
	left posterior limb of the internal capsule	0.110±0.040	0.090±0.020	0.635
	fornix	0.450±0.160	0.140±0.090	2.397
NDI	middle cerebellar peduncle	0.730±0.030	0.740±0.030	-0.333
	corpus callosum	0.500±0.050	0.530±0.050	-0.600
	right corticospinal tract	0.720±0.040	0.730±0.050	-0.221
	right cerebral peduncle	0.740±0.030	0.750±0.040	-0.282
	left cerebral peduncle	0.540±0.080	0.570±0.040	-0.476
	right internal capsule	0.600±0.040	0.660±0.050	-1.323
	left internal capsule	0.460±0.060	0.530±0.050	-1.269
	right anterior corona radiata	0.450±0.060	0.530±0.060	-1.333
	left anterior corona radiata	0.590±0.080	0.670±0.040	-1.270
	right superior corona radiata	0.560±0.060	0.630±0.050	-1.269
	left superior corona radiata	0.530±0.090	0.590±0.050	-0.827
	right posterior corona radiata	0.520±0.110	0.530±0.050	-0.118
	left posterior corona radiata	0.490±0.060	0.560±0.040	-1.377
	right posterior thalamic radiation	0.460±0.060	0.500±0.040	-0.787
	left sagittal stratum	0.380±0.030	0.400±0.030	-0.667
	right external capsule	0.440±0.030	0.470±0.030	-1.000
	left external capsule	0.520±0.090	0.530±0.050	-0.138
	right cingulum	0.420±0.040	0.440±0.030	-0.567
	fornix	0.570±0.060	0.640±0.040	-1.377
	left superior longitudinal fasciculus	0.410±0.040	0.420±0.030	-0.283

Table 3. A comparison of diffusion parameters in the white matter of the AD patients vs. the DLB subjects

	ROI	AD	DLB	Cohen's d
NDI	corpus callosum	0.491±0.053	0.517±0.060	-0.464
	left cerebral peduncle	0.747±0.033	0.775±0.050	-0.684
	left internal capsule	0.606±0.040	0.633±0.065	-0.520
	right internal capsule	0.515±0.028	0.539±0.070	-0.480
	right anterior corona radiata	0.449±0.054	0.502±0.077	-0.821
	left anterior corona radiata	0.448±0.053	0.470±0.071	-0.360
	right superior corona radiata	0.578±0.072	0.621±0.074	-0.590
	left superior corona radiata	0.557±0.055	0.573±0.077	-0.246
	right posterior corona radiata	0.520±0.097	0.540±0.071	-0.230

	right posterior thalamic radiation	0.483±0.056	0.513±0.062	-0.512
	right sagittal stratum	0.447±0.052	0.481±0.063	-0.598
	right external capsule	0.382±0.027	0.410±0.044	-0.798
	left external capsule	0.442±0.035	0.449±0.050	-0.167
	left cingulum	0.418±0.049	0.461±0.084	-0.653
	right superior longitudinal fasciculus	0.531±0.052	0.563±0.070	-0.532
	left superior longitudinal fasciculus	0.560±0.044	0.593±0.079	-0.540
	left external capsule	0.442±0.035	0.449±0.050	-0.167
ODI	genu of the corpus callosum	0.663±0.077	0.671±0.068	-0.109
	left internal capsule	0.265±0.065	0.303±0.044	-0.707
	left anterior corona radiata	0.304±0.013	0.313±0.025	-0.475
	left superior corona radiata	0.263±0.036	0.273±0.033	-0.292
	left posterior corona radiata	0.254±0.052	0.296±0.073	-0.645
	left posterior thalamic radiation	0.249±0.055	0.319±0.098	-0.845
	left sagittal stratum	0.301±0.052	0.348±0.080	-0.674
	left external capsule	0.272±0.020	0.281±0.024	-0.414
	left fornix	0.365±0.055	0.375±0.055	-0.182
	left superior longitudinal fasciculus	0.257±0.033	0.260±0.049	-0.074

Table 4. The optimal k values determined via fold-wise grid search in stratified five-fold cross-validation for sex-adjusted KNN models

group	Parameters	optimal k values
DLB＜AD	NDI	1
	ODI	5
NC＜DLB	FA	5
	MD	7
	NDI	2
	ODI	4
	VISO	5
NC＜AD	FA	5
	MD	5
	NDI	3

ODI	1
VISO	1
